# Neural Mechanisms Underlying Expectation-Guided Decision-Making

**DOI:** 10.3389/fnbeh.2022.943419

**Published:** 2022-07-01

**Authors:** Thorsten Kahnt

**Affiliations:** National Institute on Drug Abuse Intramural Research Program, National Institutes of Health, Baltimore, MD, United States

**Keywords:** decision-making, expectation, outcome-guided, inference, orbitofrontal cortex, model-based

## Introduction

Many decisions are guided by expectations about their outcomes. For instance, we may decide to visit a restaurant because we anticipate the food to be outstanding. How these expectations are represented in the brain, and how they allow us to make adaptive choices are important questions for understanding the neural basis of behavior.

Work across species has revealed brain areas that signal expected rewards (Haber and Knutson, 2010; Kahnt, 2018). This work typically focuses on neural correlates of the value of choice options (Padoa-Schioppa, 2011), that is, how desirable an option is. Activity in many brain areas, including the striatum, ventromedial prefrontal cortex and orbitofrontal cortex (OFC), is correlated with expected value. However, expected outcomes are more than their value—they have a specific identity. Even though we may equally desire pizza funghi and spaghetti arrabiata, they are not the same, and representing expectations about the identity of outcomes is important for adaptive decision-making.

In this opinion, I will summarize recent work from my lab that has shown how the lateral OFC represents expectations about specific outcomes, how these expectations are learned, and how they can be used for adaptive decision-making. Finally, I will summarize evidence that disrupting activity in OFC networks that represent specific outcome expectations impairs adaptive behavior. Together, these findings support the view that the OFC contributes to expectation-guided decision-making by enabling us to simulate the consequences of our choices.

## Neural Representations of Outcome Expectations

Recent studies have shown that the OFC represents not only expectations about the value of future outcomes but also their identity (Howard and Kahnt, 2021). For instance, in one study, we used food odors as specific rewards and selected one sweet and one savory odor for each subject that were matched in rated pleasantness (i.e., value) (Howard et al., 2015). We then lowered the concentration of the food odors to create a set of low-intensity odors, which were rated as less pleasant than the high-intensity odors. The four food odors were then paired with different visual stimuli, such that each odor was reliably predicted by a different symbol. Finally, subjects were presented with these symbols while undergoing functional magnetic resonance imaging (fMRI). Multi-voxel pattern analysis (Kahnt, 2018) to the fMRI responses evoked by the symbols revealed that activity patterns in the lateral OFC, anterior cingulate cortex, and hippocampus differentiated between the two expected food odors, whereas activity patterns in the medial OFC represented the value of the odors, independent of their identity. These findings are in line with other work from our lab (Howard and Kahnt, 2017) as well as with studies showing that activity patterns in the lateral OFC represent values that are tied to specific reward categories, whereas activity in the medial OFC is independent of reward category (Mcnamee et al., 2013).

## Learning of Outcome Expectations

Outcome expectations are based on associations between predictive stimuli and rewards, and these associations need to be learned and updated through experience. Work in non-human primates has shown that dopamine neurons in the midbrain contribute to learning the value of rewards by signaling reward prediction errors, or the difference between received and expected rewards (Schultz et al., 1997). We hypothesized that midbrain activity encodes a similar signal for identity prediction errors, which may be used for learning reward identity expectations.

In one experiment, hungry subjects were presented with visual symbols that predicted one of two preference-matched food odors (e.g., strawberry or potato chips) in either low or high intensity (Howard and Kahnt, 2018). As in previous studies, subjects reported a higher preference for the high-intensity odors, but there was no preference difference between the sweet and savory food odors. After a number of trials of receiving the predicted odor, either the identity (e.g., subjects expected strawberry but received equally-preferred potato chips) or the intensity (e.g., subjects expected potato chips in low intensity but received the preferred high-intensity odor) of the odor was unexpectedly changed. fMRI activity in the midbrain showed signatures of value-based prediction errors, increasing when subjects received the more preferred high-intensity odor after expecting the less preferred low-intensity odor. However, activity in the same midbrain region also increased when subjects received strawberry after expecting potato chips, in line with the signaling of value-neutral identity prediction errors. Importantly, value- and identity-based prediction errors were found in the same part of the midbrain and were correlated, suggesting that they may originate from the same neural population. Similar findings have been observed in a study that recorded activity from dopamine neurons in rats (Takahashi et al., 2017), as well as in other human imaging studies (Boorman et al., 2016; Schwartenbeck et al., 2016; Suarez et al., 2019).

A question that follows is whether midbrain identity prediction errors actively shape identity learning in downstream areas, or whether they merely act as a permissive gating (i.e., salience) signal to direct attention and boost learning (Bromberg-Martin et al., 2010). We addressed this question, reasoning that if identity prediction errors conveyed salience information without providing specific information, there should be no difference between the midbrain response to reward B when A was predicted and the midbrain response to reward A when B was predicted. In contrast, if identity prediction errors actively shape learning in downstream targets, they should contain specific information such that midbrain responses differ between these two cases. In line with the latter idea, we found that midbrain fMRI patterns in humans and dopamine ensemble responses in rats contain information about the specific identity of the error (Stalnaker et al., 2019), suggesting they could directly update identity expectations in downstream areas, such as OFC.

Indeed, we found that the magnitude of identity prediction error response in the midbrain was correlated with how much identity expectations in the lateral OFC changed after an identity error (Howard and Kahnt, 2018). This suggests that identity expectations in the lateral OFC are updated through a mechanism that involves identity prediction errors in the dopaminergic midbrain.

## Using Expectations for Inference

In many cases, we can learn the expected value of choice options through direct experience. For instance, we can learn the value of an item on a restaurant menu by ordering it. However, for many other decisions in life, we simply have not had the opportunity to directly learn values in this way. This especially applies to decisions that are less frequently or only indirectly experienced, like deciding to try out a new restaurant or whether to visit a new country. Also, the values we have learned from previous decisions may have changed since we last made that choice, and using these old values would lead to maladaptive decisions. In these situations, value expectations need to be computed by mentally simulating or inferring the value of the option based on incomplete information. Specific outcome expectations allow us to do this because they are part of a model of the relevant environment which we can use to simulate the consequences of our actions.

Such simulations can be studied in the devaluation task. In a typical experiment, subjects first learn to associate different sensory cues with different foods, e.g., M&Ms and peanuts (Rudebeck et al., 2013; Murray et al., 2015; Reber et al., 2017). After one of the rewards is devalued by feeding the food to satiety, subjects can make choices between the sensory cues. To access the current value of the choice option, subjects must simulate what outcome they will receive by making a particular choice and infer its current value. This allows them to avoid selecting the cue that predicts the devalued outcome. In contrast, if they use the previously learned value, they will make choices that result in both the valued and the devalued outcome.

We have used transcranial magnetic stimulation (TMS) to test whether outcome identity expectations represented in the lateral OFC are necessary for adaptive responding in the devaluation task (Howard et al., 2020). Hungry participants first learned associations between visual symbols and sweet or savory food odors and were then allowed to make choices between these symbols. Stimulation coordinates in the lateral PFC were selected for each participant based on resting-state fMRI connectivity with lateral OFC. After a session of continuous theta burst stimulation (cTBS), which has inhibitory after-effects lasting for 50–60 min (Huang et al., 2005), or sham stimulation, subjects ate a meal that was matched to either the sweet or the savory food odor. After this devaluation procedure, subjects could again make choices between the cues. Targeting the lateral OFC with cTBS had profound effects on subjects' choices after the meal. Whereas, subjects in the sham group adaptively stopped selecting symbols that predicted the devalued odor, subjects in the cTBS group continued to select these stimuli. This shows that OFC activity is required for using specific outcome expectations for making inferences about the current value of choice options.

A different type of inference can be probed in the sensory preconditioning task (Brogden, 1939; Hoffeld et al., 1960). In this task, subjects first learn associations between sensory stimuli A and B, and C and D (A → B, C → D). Then, the second cue of each pair (B and D) is paired with either a reward or no reward (B → reward, D → no reward). Finally, responses to all stimuli (A, B, C, and D) are probed. Humans and other animals show stronger responding to stimulus A compared to stimulus C in this final test (Sadacca et al., 2016; Sharpe et al., 2017; Wang et al., 2020b). This pattern of responding is compatible with the idea that subjects mentally step through the associations A → B and B → reward to infer that A → reward.

Activity in the OFC correlates with learning of the stimulus-stimulus associations during the initial learning phase (Sadacca et al., 2018; Wang et al., 2020b), suggesting that the OFC represents the associative structure of the task. In other words, stimulus-stimulus associations appear to be represented in the same way as associations between a sensory stimulus and a food reward. Moreover, OFC is critical for using these associations to perform mental simulations. Pharmacological inactivation of the lateral OFC in rats (Jones et al., 2012) as well as cTBS targeting the lateral OFC network in humans before the final phase of the sensory preconditioning task impairs responding to cue A, without affecting responding to cue B (for which subjects had directly learned the stimulus-outcome associations) (Wang et al., 2020a). Thus, just like neural representations of specific outcome expectations, representations of stimulus-stimulus associations in the lateral OFC network are critical for making mental simulations required for adaptive decision-making.

## Discussion

The work described above outlines the neural mechanisms underlying expectation-guided decision-making. In brief, the OFC represents expectations about specific outcomes, and these expectations are learned through an error-based mechanism that involves the dopaminergic midbrain. The same networks that represent outcome expectations also represent expectations about future events, even if they do not possess any value. Of note, while we often make decisions between options with outcomes that belong to very different categories, our experiments used outcomes from the same reward category (i.e., food). This can be considered a stronger test of the outcome-specific coding hypothesis, because differences in neural responses to different reward categories may not only reflect outcome-specific coding but also different preparatory or consummatory reward responses. Thus, results from within category experiments are likely to generalize to across category settings. Indeed, previous work on neural representations of different reward categories has revealed comparable findings (Levy and Glimcher, 2011; Mcnamee et al., 2013; Gross et al., 2014).

Neural representations of specific outcomes enable us to perform mental simulations that are required for adaptive behavior in novel situations or when the value of an outcome has changed since we last made that decision. In other words, these representations allow us to flexibly assign value or meaning to expected outcomes in order to guide our decisions. Together, the findings discussed here are compatible with the view that the OFC network contributes to decision-making by representing a model of the environment, which enables us to make flexible inferences about the outcomes of our decisions.

## Author Contributions

The author confirms being the sole contributor of this work and has approved it for publication.

## Funding

This study was funded by Dr. Rüdiger Seitz, *via* the Volkswagen Foundation, Siemens Healthineers, and the Betz Foundation. The author was supported by the Intramural Research Program at the National Institute on Drug Abuse, and the expressed opinions are the author's own and do not reflect the view of the NIH/DHHS. Siemens Healthineers was not involved in the study design, collection, analysis, interpretation of data, the writing of this article or the decision to submit it for publication.

## Conflict of Interest

The author declares that the research was conducted in the absence of any commercial or financial relationships that could be construed as a potential conflict of interest.

## Publisher's Note

All claims expressed in this article are solely those of the authors and do not necessarily represent those of their affiliated organizations, or those of the publisher, the editors and the reviewers. Any product that may be evaluated in this article, or claim that may be made by its manufacturer, is not guaranteed or endorsed by the publisher.
